# Robot-assisted vs laparoscopic lateral transabdominal adrenalectomy: a propensity score matching analysis

**DOI:** 10.1007/s00464-022-09663-3

**Published:** 2022-10-03

**Authors:** Carmela De Crea, Francesco Pennestrì, Nikolaos Voloudakis, Luca Sessa, Priscilla Francesca Procopio, Pierpaolo Gallucci, Rocco Bellantone, Marco Raffaelli

**Affiliations:** 1 U.O.C. Chirurgia Endocrina e Metabolica, Centro Dipartimentale di Chirurgia Endocrina e dell’Obesità, Fondazione Policlinico Universitario Agostino Gemelli IRCCS, Rome, Italy; 2 Centro di Ricerca in Chirurgia Endocrina e dell’Obesità, Università Cattolica del Sacro Cuore, L.go A. Gemelli 8, 00168 Rome, Italy; 3 Centro Malattie Endocrine e Obesità, Fondazione Gemelli Giglio Cefalù, Cefalù, Palermo, Italy

**Keywords:** Laparoscopic adrenalectomy, Lateral transabdominal adrenalectomy, Robot-assisted adrenalectomy, Postoperative complications, Outcomes

## Abstract

**Background:**

Laparoscopic adrenalectomy (LA) is the gold standard treatment for adrenal lesions. Robot-assisted adrenalectomy (RAA) is a safe approach, associated with higher costs in absence of clear-cut benefits. Several series reported some advantages of RAA over LA in challenging cases, but definitive conclusions are lacking. We evaluated the cost effectiveness and outcomes of robotic (R-LTA) and laparoscopic (L-LTA) approach for lateral transabdominal adrenalectomy in a high-volume center.

**Methods:**

Among 356 minimally invasive adrenalectomies (January 2012–August 2021), 286 were performed with a lateral transabdominal approach: 191 L-LTA and 95 R-LTA. The R-LTA and L-LTA patients were matched for lesion side and size, hormone secretion, and BMI with propensity score matching (PSM) analysis. Postoperative complications, operative time (OT), postoperative stay (POS), and costs were compared.

**Results:**

PSM analysis identified 184 patients, 92 in R-LTA and 92 in L-LTA group. The two groups were well matched. The median lesion size was 4 cm in both groups (*p* = 0.533). Hormonal hypersecretion was detected in 55 and 54 patients of R-LTA and L-LTA group, respectively (*p* = 1). Median OT was significantly longer in R-LTA group (90.0 vs 65.0 min) (*p *< 0.001). No conversion was registered. Median POS was similar (4.0 vs 3.0 days in the R-LTA and L-LTA) (*p* = 0.467). No difference in postoperative complications was found (*p* = 1). The cost margin analysis showed a positive income for both procedures (3137 vs 3968 € for R-LTA and L-LTA). In the multiple logistic regression analysis, independent risk factors for postoperative complications were hypercortisolism (OR = 3.926, *p* = 0.049) and OT > 75 min (OR = 8.177, *p* = 0.048).

**Conclusions:**

The postoperative outcomes of R-LTA and L-TLA were similar in our experience. Despite the higher cost, RAA appears to be cost effective and economically sustainable in a high-volume center (60 adrenalectomies/year), especially if performed in challenging cases, including patients with large (> 6 cm) and/or functioning tumors.

**Graphical abstract:**

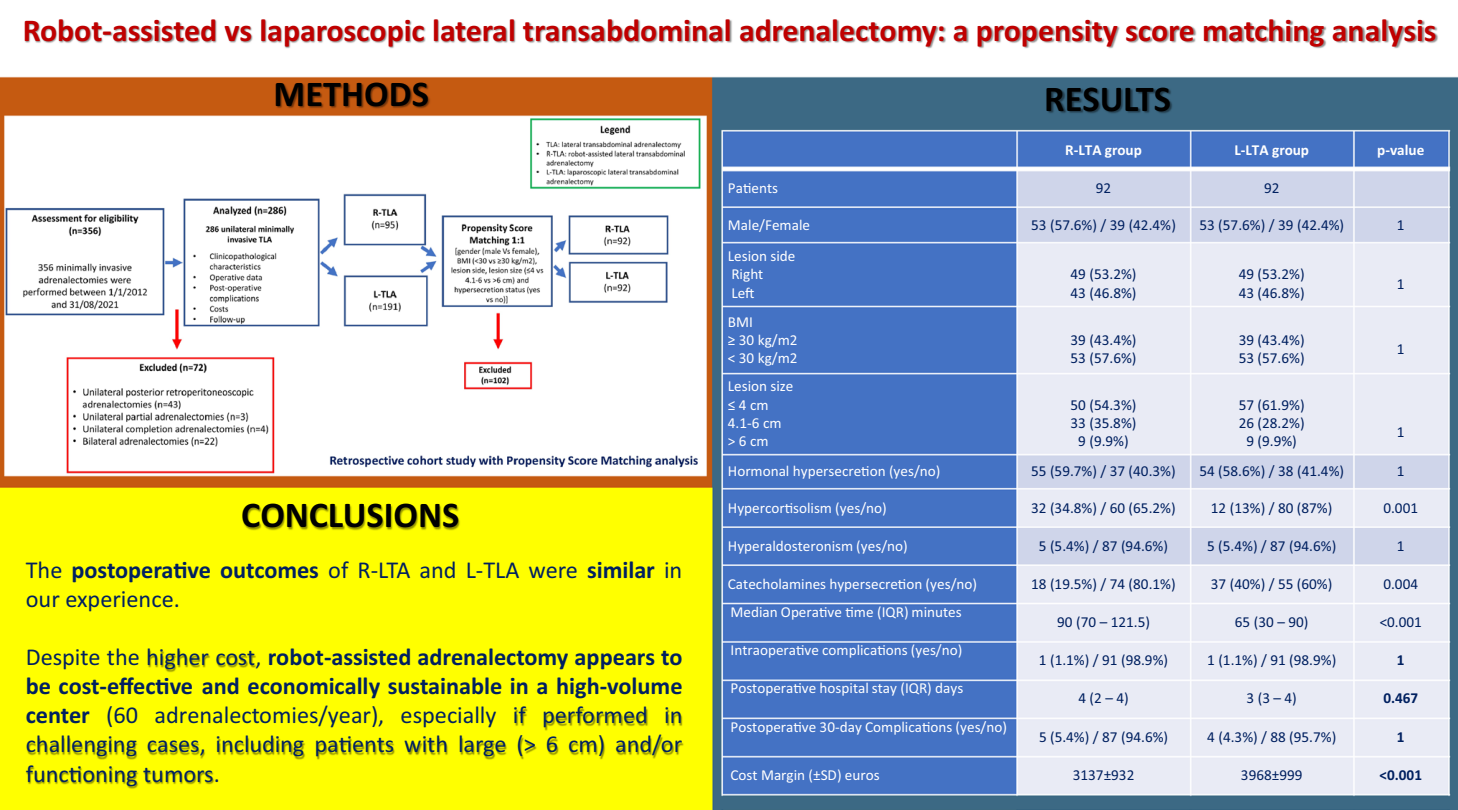

**Supplementary Information:**

The online version contains supplementary material available at 10.1007/s00464-022-09663-3.

## Background

Since the initial description [1] and standardization [2, 3] of laparoscopic lateral transabdominal adrenalectomy (LTA), its application in the clinical practice exponentially increased and quickly became the gold standard treatment for adrenalectomy [4–6].

Although randomized controlled studies comparing the laparoscopic adrenalectomy (LA) and the open approach are lacking, the benefits of minimally invasive surgery such as less postoperative pain, shorter hospital stay and recovery time, and lower complication rates were clearly demonstrated [7–14].

The introduction of robotic technology [15, 16] highlighted several known limitations of laparoscopy, such as unstable operating field, orientation errors due to camera holding, restrictions in range of movement, natural hand fatigue, flapping tremors, and 2-dimensional operative view [17].

Thereafter, the widespread diffusion of robotic platforms has led to the development and standardization of robot-assisted adrenalectomy (RAA) [18]. RAA has been shown to be feasible and safe in several studies and became an option for adrenalectomy in several centers [18–26]. The perceived advantages of RAA include improved ergonomics, stereoscopic vision, tremor filtration, greater range of motion within the operative field, and articulation of the working arm [27, 28], potentially resulting in an ameliorated surgical dexterity and theoretically, maximizing the surgical efficiency of conventional LA.

Despite these subjective advantages, the role of robotic surgery is still not precisely addressed [20–28].

Several variables, such as Body Mass Index (BMI) [19, 22], previous abdominal surgery, and tumor size [29] have been evaluated in different clinical settings, in order to figure out whether the robot-assisted LTA is preferable over laparoscopic LTA in selected challenging patients and/or in complex adrenal lesions. However, to date, no unequivocal benefit from the use of the RAA has been found [26], while increased costs still represent a drawback [19, 26, 29–33].

More recently, a large retrospective multicenter analysis from the European Surgical Registry EUROCRINE showed that RAA compared with laparoscopic LTA resulted in a lower complication rate and shorter postoperative hospital stay [34]. However, further studies are required in order to validate these conclusions.

With that purpose, we performed a retrospective evaluation of the cost effectiveness and outcomes of robotic versus laparoscopic approach for LTA in a high-volume endocrine referral center.

## Material and methods

Laparoscopic adrenalectomy was introduced in our clinical practice in 1998 while robot-assisted adrenalectomy via lateral transabdominal access in 2012. In our Institution that is a tertiary referral center for endocrine surgery, data from all patients scheduled for adrenalectomy are prospectively collected in a specifically designed and de-identified database.

### Study population

All patients who were scheduled for minimally invasive unilateral LTA between January 2012 and August 2021 were candidates for inclusion. Among 356 patients who underwent minimally invasive unilateral adrenalectomy (intention-to-treat analysis), 286 adrenalectomies were performed with lateral transabdominal approach and were included in the study. Patients who underwent bilateral or subtotal adrenalectomy, posterior retroperitoneoscopic adrenalectomy, open adrenalectomy, and those with concomitant procedures at the time of adrenalectomy were excluded from the analysis. Based on the access route, laparoscopic or robot-assisted, patients were divided into two groups: Robot-assisted Lateral Transabdominal Adrenalectomy group (R-LTA) and Laparoscopic Lateral Transabdominal Adrenalectomy group (L-LTA).

Baseline patients’ characteristics included gender, age, and body mass index (BMI). Preoperative characteristics included hormonal status and tumor side and size. Operative parameters consisted of surgical approach, operative time, intraoperative complication, and conversion rate. Postoperative parameters included histopathology, length of hospital stay, early complications, readmission, and mortality.

The preoperative workup included clinical, biochemical, and radiological evaluation according to international society guidelines [35–37]. In patients with functional or suspected malignant adrenal lesions, further molecular, nuclear medicine, and radiological imaging studies were performed according to the specific clinical scenario [38, 39]. The specific preoperative protocol for patients with catecholamine-secreting neoplasms is described in the supplementary materials.

Follow-up evaluation was obtained by outpatient consultation or telephone contact. For this study, follow-up evaluation ended on 31st March 2022.

To account for the effect of possible confounders on outcomes, the R-LTA and L-LTA patients were matched for lesion side and size, hormonal secretion status, and BMI with propensity score matching (PSM) analysis.

Additionally, a cost analysis of robot-assisted vs laparoscopic LTA was performed.

The study was submitted and approved by the ethical board of our Institution (Identification Study Number: 4853; Protocol Study Number: 0019329/22).

### Study end-points

The primary endpoint was to compare the robotic vs laparoscopic approach for LTA in terms of complication rate. The secondary endpoint was to compare the two approaches in terms of operative time and hospital stay.

### Definitions

The operative time is defined as the interval from incision to wound closure (skin to skin). The severity of postoperative complications was graded according to the Clavien-Dindo classification [40]. Intraoperative complications were defined as all the events that could potentially cause injury and require unplanned surgical maneuvers. Postoperative complications were defined as any event altering the normal postoperative course and/or delaying discharge, occurring until the 30th postoperative day. Mortality was defined as any intraoperative or postoperative death within 30 days of surgery. Follow-up time is defined as the time interval between the date of the surgical procedure and the date of the last follow-up examination.

Locoregional recurrence is defined as recurrence of disease at the surgical site, while recurrence in other anatomical regions is defined as progression of systemic disease. The time of locoregional recurrence is defined as the time interval between the date of surgery and the date of recurrence.

The costs evaluation was performed per each patient by our administrative service. The overall detail of costs was collected in an institutional administrative database. In our country (Italy), the reimbursement for adrenalectomy is €7695, and it is the same for laparoscopic and robotic surgeries (flat reimbursement). So, the hospital did not receive extra money in case of robotic procedures. Moreover, the Diagnostic-Related Group (DRG) reimbursement for adrenalectomy is the same even in case of postoperative complications as opposed to other surgical procedures. In our hospital, the operating room staff is paid a fixed salary. With this purpose, our economists performed different cost analysis and in particular a combination of bottom-up micro-costing and top-down gross costing. To delve deeper, the cost evaluation for operating room cost was performed using top-down gross costing and included anesthesia (*n* = 1), surgery (*n* = 2), and scrub nurse (*n* = 2) professional’s costs, electricity, and sterile water costs. The cost evaluation of hospital stay is composed by a bottom-up micro-costing for drugs, radiological, or biochemical exams, and a top-down gross costing for surgery and nurse professional’s costs, and accommodation costs. The cost evaluation for medical devices was performed using bottom-up micro-costing. Overall costs were subtracted from the DRG reimbursement for adrenalectomy in order to calculate the operating margins for robotic and laparoscopic approach for LTA [31].

### Surgical techniques

All procedures were performed by an expert endocrine surgeon (R.B. and M.R). Both the operating surgeons performed more than 100 minimally invasive adrenalectomy before the study period. On the basis of current literature, we can assume that they have acquired advanced skills for this procedure [41].

Informed consent was obtained prior to operation in all cases. The choice of the surgical approach was taken on the basis of the patient and lesion characteristics and of the surgeon’s preference [42]. The surgical techniques (robotic and laparoscopic approach for LTA) were previously reported in detail [43–46]. One hundred mg intravenous hydrocortisone was administered intraoperatively after the dissection of the main adrenal vein.

### Postoperative protocol

The postoperative patients’ protocol is described in the supplementary materials.

### Statistical analysis

Propensity score matching was obtained with the “1:1 nearest neighbour” matching method (discard = both groups, caliper = 0.2). Type of surgical approach (laparoscopic or robotic) was entered into the regression model of the propensity score as the binary treatment variable. The following covariates, estimated to be important for postoperative complications, were included into the analysis: gender (male Vs female), BMI (< 30 vs ≥ 30 kg/m^2^), lesion side, lesion size (≤ 4 vs 4.1–6 vs > 6 cm), and hypersecretion status (yes vs no).

Baseline characteristics, and operative and postoperative variables were compared using a bivariate analysis. Normal distribution was assessed using the Shapiro-Wilks test. Fisher's exact test and Chi-square test were used to compare categorical variables. Continuous variables were expressed as median (interquartile range, IQR). Odds ratios (OR) were expressed as value (95% interval of confidence). We used paired sample t test or Wilcoxon test to compare continuous variables, depending on data distribution of the analyzed population. Backward stepwise logistic regressions were performed in order to evaluate the potential risk factors. At each step, the variable that had the lowest correlation with the outcome was removed with an elimination criterion set at *p* > 0.100 and a threshold of *p* = 0.1 to set a limit on the total number of variables included in the final model. Only variables with a *p* < 0.2 on univariate analysis or clinical importance were entered in the model.

In the cost analysis, although variables were non-parametric, we report them as means ± standard deviation (95% interval of confidence) to conform to the established economic reports methodology. Basic demographic and clinical data were collected through review of patients’ charts and electronic databases. Statistical analysis and PSM were conducted with SPSS 22.0 software for Windows (SPSS Inc, Chicago, III). All analyses were two tailed, and the threshold for statistical significance was set at *p* < 0.05.

## Results

**Fig. 1:**
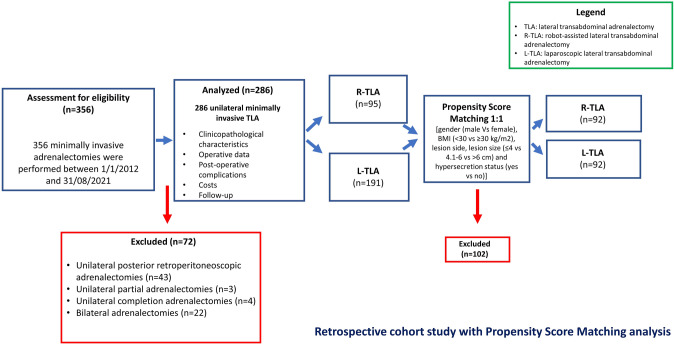
Study patients’ flowchart diagram

During the study period (January 2012 and August 2021), 356 minimally invasive adrenalectomies were performed. A total of 286 (80.3%) patients were scheduled for LTA and were included in the study. All patients initially scheduled for laparoscopic or robotic LTA underwent to the planned procedures. Laparoscopic approach was performed in 191 (66.8%) cases, while robot-assisted approach was performed in 95 (33.2%) cases. After PSM, the study population consisted of 184 patients: 92 in the R-LTA and 92 in the R-LTA group, respectively (overall balance test: chi-square: 0.371, *p* = 0.985; multivariate imbalance measure L1 before match 0.370 and after matching 0.227). In Fig. 1, a study patients’ flowchart diagram was reported. The characteristics of the study’s population are shown in Table 1. There were 106 females and 78 males. The median age was 54 (46.25–64) years and the median preoperative BMI was 27 (23–33) kg/m^2^. Overall, the median operative time was 75 (40.5–109.5) minutes and the median postoperative hospital stay was 4 (3–5) days. In the present series, no readmission after discharge nor conversions (either from laparoscopic to open surgery, or from robot-assisted to laparoscopic/open surgery) were registered. Nine patients (4.9%) develop minor postoperative complications. Thirty-day mortality rate was zero.

**Table 1:** Characteristics of the study’s population and comparative analysis between R-LTA and L-LTA after propensity matching score

	Total	R-LTA group	L-LTA group	*p* value*
Number of patients	184	92	92	
Male/female	106 (57.8%) / 78 (42.4%)	53 (57.6%) / 39 (42.4%)	53 (57.6%) / 39 (42.4%)	1
Median age (IQR) years	54 (46.25–64)	55 (45.25–64.75)	54 (47–61)	0.610
Lesion side				
Right	98 (53.3%)	49 (53.2%)	49 (53.2%)	1
Left	86 (46.7%)	43 (46.8%)	43 (46.8%)	
Median BMI (IQR) kg/m^2^	27 (23–33)	27 (22–34.75)	27.5 (23–32.75)	0.641
BMI				
≥ 30 kg/m^2^	78 (42.4%)	39 (42.4%)	39 (42.4%)	1
< 30 kg/m^2^	106 (57.6%)	53 (57.6%)	53 (57.6%)	
Median lesion size (IQR) cm	4 (3–5)	4 (3–5.57)	4 (3–5.50)	0.533
Lesion size				1
≤ 4 cm	107 (58.2%)	50 (54.3%)	57 (61.9%)	
4.1–6 cm	59 (32.1%)	33 (35.8%)	26 (28.2%)	
> 6 cm	18 (9.8%)	9 (9.9%)	9 (9.9%)	
Hormonal hypersecretion (yes/no)	109 (59.2%) / 75 (40.8%)	55 (59.7%) / 37 (40.3%)	54 (58.6%) / 38 (41.4%)	1
Hypercortisolism (yes/no)	44 (23.9%) / 140 (76.1%)	32 (34.8%) / 60 (65.2%)	12 (13%) / 80 (87%)	0.001
Hyperaldosteronism (yes/no)	10 (5.4%) / 174 (94.6%)	5 (5.4%) / 87 (94.6%)	5 (5.4%) / 87 (94.6%)	1
Catecholamines hypersecretion (yes/no)	55 (29.9%) / 129 (70.1%)	18 (19.5%) / 74 (80.1%)	37 (40%) / 55 (60%)	0.004
Median operative time (IQR) minutes	75 (40.5–109.5)	90 (70–121.5)	65 (30–90)	< 0.001
Intraoperative complications (yes/no)	2 (1.1%) / 182 (98.9%)	1 (1.1%) / 91 (98.9%)	1 (1.1%) / 91 (98.9%)	1
Postoperative hospital stay (IQR) days	4 (3–5)	4 (2–4)	3 (3–4)	0.467
Postoperative 30th day Complications (yes/no)	9 (4.9%) / 175 (95.1%)	5 (5.4%) / 87 (94.6%)	4 (4.3%) / 88 (95.7%)	1
Histology				0.016
Benign lesions	109 (59.2%)	63 (68.5%)	46 (50%)	
Pheochromocytomas	69 (31.5%)	20 (21.7%)	39 (42.4%)	
Adrenocortical carcinomas	7 (4.4%)	3 (3.3%)	4 (4.4%)	
Metastasis	9 (4.9%)	6 (6.5%)	3 (3.3%)	
Follow-up time (IQR) months	61 (33–85)	83 (68.75–98.75)	34 (22.25–50.50)	< 0.001
Locoregional recurrence (yes/no)	2 (1.1%) / 284 (98.9%)	1 (1.1%) / 91 (98.9%)	1 (1.1%) / 91 (98.9%)	1

^a^*p* values refer to comparison between R-TLA group and L-TLA group

In Table 1, the comparative analysis between R-LTA and L-LTA is reported. The two groups were comparable in terms of gender distribution, age, BMI, and lesion side and size.

The type of hypersecretion status was significantly different between the two groups, with more cases of hypercortisolisms in the R-LTA group (32 in R-LTA vs 12 in L-LTA, *p* = 0.001) and a significant predominance of catecholamine hypersecretion in the L-LTA group (18 in R-LTA vs 37 in L-LTA, *p* = 0.004).

Intraoperative complications were similar between the two groups (1 in R-LTA vs 1 in L-LTA, *p* = 1). Two cases of intraoperative bleeding from the inferior vena cava were managed endoscopically, one in each group. Both patients had a non-secreting 5 cm right adrenal lesion.

The median operative time was longer in R-LTA, 90 (70 – 121.5) min vs 65 (30–90) min, respectively, in R-LTA and in L-LTA, (*p* < 0.001) (Table 1). However, in the subgroup analysis considering BMI (≥ 30 and < 30), lesion size (≤ 4 cm, 4.1–6 cm, > 6 cm) and hypersecretion status (non-secreting lesions vs hyperaldosteronism, hypercortisolism, catecholamine hypersecretion) (Table 2), the operative time was similar between the two groups for patients with BMI ≥ 30 kg/m^2^ (*p* = 0.085), lesion size > 6 cm (*p* = 0.620), and hypersecretion of aldosterone (*p* = 0.841), catecholamines (*p* = 0.635), and cortisol (*p* = 0.545) (Table 2). Delving deeper into factors affecting the operative time in each group, by performing a backward logistic regression for potential risk factors, we observed that in the L-LTA group, hypercortisolism (OR 3.871, 95% CI: 0.966–15.544, *p* = 0.041) and a lesion size > 6 cm (OR 4.516, 95% CI: 0.876–23.280, *p* = 0.048) were independent risk factors for longer operative time (see the relative table in supplementary materials). This did not apply in the R-LTA group, where no statistically significant risk factors leading to longer operative time were found.

**Table 2:** Sub-group univariable analysis for median operative time

Sub-groups	Median operative time (IQR), minutes		
	R-LTA group	L-LTA group	p value
BMI			
BMI < 30 kg/m^2^	92 (57–141.25)	65 (45–89.5)	< 0.001
BMI ≥ 30 kg/m^2^	80 (70–102.5)	70 (55–115)	0.085
Lesion size			
≤ 4 cm	90 (70–122)	65 (45–90.5)	< 0.001
4.1–6 cm	85 (50–120)	65 (46–95.5)	< 0.001
> 6 cm	90 (75–117.5)	89 (65–94)	0.620
Nonsecreting lesions	100 (70–130)	64 (42.5–89.5)	< 0.001
Secreting lesions			
Hyperaldosteronism	75 (43.5–101)	70 (65–95.5)	0.841
Hypercortisolism	90 (68.75–100)	75 (65.50–125)	0.545
Catecholamines hypersecretion	95 (77.5–114.5)	75 (32.90–114.75)	0.635

No significant differences were found between the two groups in terms of postoperative complications (5 in R-LTA vs 4 in L-LTA, respectively, *p* = 1) (Table 3). All registered complications were grade II in the Clavien-Dindo scale (Table 3).

**Table 3:** Postoperative early complications

	R-LTA group	L-LTA group
Atrial fibrillation		
Clavien–Dindo grade II	1 (1.1%)	1 (1.1%)
Pneumonia		
Clavien–Dindo grade II	1 (1.1%)	1 (1.1%)
Superficial surgical site infection		
Clavien–Dindo grade II	1 (1.1%)	2 (2.2%)
Deep surgical site infection		
Clavien–Dindo grade II	2 (2.2%)	0 (0%)

The postoperative hospital stay was 4 (2–4) days and 3 (3–4) days (*p* = 0.467), respectively, in R-LTA and L-LTA.

To explore potential risk factors for postoperative complications and postoperative hospital stay > 4 days, we performed a backward logistic regression analysis (Table 4). Hypercortisolism and operative time > 75 min were identified as significant risk factors for postoperative complications (Table 4).

**Table 4:** Risk factors for increased postoperative complications and hospital stay (multivariate backward stepwise logistic regression analysis)

	OR	95% CI	*p*-value
Postoperative 30 day complications			
Hypercortisolism	3.926	0.979–15.745	0.048
Operative time > 75 min	8.177	0.990–65.516	0.049
Postoperative hospital Stay > 4 days			
Hypercortisolism	4.949	2.044–11.982	< 0.001
Operative time > 75 min	3.887	1.972–7.662	< 0.001

Histopathology results yielded 7 cases of adrenocortical carcinoma (3 in the R-LTA and 4 in the L-LTA, respectively) and 9 cases of adrenal metastases (6 in the R-LTA and 3 in the L-LTA, respectively).

The median follow-up time of the entire series was 61 (33 – 85) months and differed significantly between the two groups, 83 (68.75 – 98.75) months in R- LTA vs 34 (22.25 – 50.50) months in L-LTA (*p* < 0.001) (Table 1).

Overall, the rate of locoregional recurrence was similar between the two groups (1 case in R-LTA Vs 1 case in L-LTA, *p* = 1). More specifically, the locoregional recurrence in the L-LTA group presented at 24 months and concerned a patient with a 9 cm incidentaloma and a final pathology of adrenocortical carcinoma; the locoregional recurrence in the R-LTA group concerned a patient with a 3 cm melanoma metastasis, which recurred at 54 months. In metastatic lesions, five cases of systemic disease progression were registered. Three of those cases concerned adrenal metastasis originating from renal cancer (two in R-LTA and one in L-LTA), one from colon cancer (L-LTA) and one from melanoma (R-LTA). In the last case, the patient died 12 months post-operatively.

Finally, the cost margin analysis showed a positive income for both procedures: 3137 ± 932 (2429–3849) vs 3968 ± 999 (3077–4179) €, respectively, for R-LTA and L-LTA (*p* < 0.001).

## Discussion

The present retrospective cohort study reports a comparative analysis between robot-assisted and laparoscopic adrenalectomy performed at a high-volume endocrine referral center from January 2012 to August 2021. The overall adrenal caseload of our Institution during the study period was 446 adrenalectomies with an annual volume of adrenalectomies of approximately 60 cases in the last three years.

Concerning the primary outcome of the study, we found comparable intraoperative (1.1% vs 1.1% for R-LTA and L-LTA, respectively) and postoperative complication rates (5.4% vs 4.1%, for R-LTA and L-LTA respectively) between the two groups. All postoperative complications we observed were medical (Clavien-Dindo grade II) and potentially related to patients features and preoperative diagnosis (e.g., hypercortisolism).

Similarly to the majority of studies on this topic [23, 24, 34, 47–54], our experience confirms that robot-assisted adrenalectomy is a safe technique with acceptable perioperative complications rate.

Most of the previous reports and meta-analysis exhibited similar results in terms of operative complications between these two approaches [24, 47–54]. Brandao et al. [23] exhibited a not significant trend favoring robot-assisted adrenalectomy for postoperative complications in an analysis including 600 adrenalectomies performed mostly by the transperitoneal route (in 72.5% of robot-assisted and in 75.5% of laparoscopic adrenalectomy, respectively). Interestingly, the study groups significantly differed for BMI, which was higher in the laparoscopic group [23]. More recently, Vatansever et al. [34], in a large multicenter study comparing robot-assisted versus conventional laparoscopic adrenalectomy reported significantly lower complications rate in the robot-assisted group (16.5% vs 1.6%). However, in the subgroup analysis considering only centers performing either robot-assisted or laparoscopic TLA, the complications rate was comparable (1.6% Vs 2.7% of complication rate, respectively, for robot-assisted and laparoscopic adrenalectomy) [34].

Our analysis identified hypercortisolism and operative time > 75 min (the median operative time of the entire series) as risk factors for developing postoperative complications, independently from the surgical approach applied (RAA and LA) (see table 5). This is in accordance with other publications that reported increased rates of postoperative complications in patients affected by Cushing syndrome related to their clinical condition [55–59]. It is noteworthy that in the present series, the complication rate between R-LTA and L-LTA was similar, despite the significantly higher number of Cushing patients in R-TLA group (32 in R-LTA vs 12 in L-LTA, *p* = 0.001).

In the present study, operative time was longer in R-LTA compared to the L-LTA (90 vs 65 min). It is acknowledged that the operative time for RAA, at the initial phases of application, is longer compared to LA [60]. As a matter of fact, some studies specifically underlined that the docking step is responsible for a significant increase of operative time in RAA [17]. Moreover, several variables (robotic-dedicated operative room, completing the preparations of the robotic platform during the anesthesia and familiarity of the surgical team with robotic surgery) have a significant impact on operative time [60]. On the other hand, extensive experience with laparoscopic surgery and previous exposure to robotic procedures are able to significantly reduce the learning curve of RAA [20, 60].

The application of robotic technology to more challenging patients and complex tumors seems to be favorable in terms of operative time. This is quite evident in our subgroup analysis concerning the operative time (see Table 2). Indeed, by analyzing separately obese patients (BMI ≥ 30 kg/m^2^), secreting lesions (hypercortisolism, hyperaldosteronism, catecholamines hypersecretion), and lesion size > 6 cm, the operative time between the two groups was comparable. Delving deeper in the data, we observed that in such more complex cases, the operative time of robot-assisted adrenalectomy remained substantially unchanged compared to the 90 min of the entire R-LTA group, while the operative time of the laparoscopic adrenalectomy increased (see Table 2). Similar results are deductible from different studies. In a case–control study, RAA showed potential benefits compared to LA, especially in patients with tumor size ≥ 6 cm, BMI ≥ 30 kg/m^2^, and previous abdominal surgery [25]. From a theoretical point of view, if the interposition of the surgeon–computer interface can maximize the efficiency of the surgical procedure, RAA would be more appropriate in this scenario. Similar conclusions were reported by Vatansever et al. [34] and are in line with our results. Indeed, in our analysis, no risk factors leading to longer operative time for R-LTA were identified. On the contrary, in L-LTA, several risk factors seem able to prolong the operative time. By using a backward logistic regression analysis, we identified hypercortisolism and lesion size > 6 cm as risk factors for longer operative time for L-TLA. In the present study, the median postoperative hospital stay was comparable between the two groups, even though more patients with hypercortisolism were present in the R-LTA group. Indeed, hypercortisolism was found as a risk factor for longer postoperative hospital stay (see table 5). Other authors reported shorter postoperative hospital stay in favor of RAA but with highly variable results [24, 26, 34, 53, 54, 61]. Since hospital stay may be affected by differences in health care reimbursement systems, patients’ proximity to the referral center and cultural expectations.

In our study, the two groups were comparable in terms of local recurrences. However, we acknowledge that this study was not designed to address this specific issue.

The increased costs of robotic-assisted adrenalectomy remain one of the main hurdles for widespread application. According to our previous analysis [31], both procedures in this study presented a positive cost margin. Obviously, L-LTA was associated with a higher mean cost margin over R-LTA. This is attributed almost solely on the different medical devices costs [31]. Brunaud et al. [19] pointed out that the cost of RAA was 2.3 times higher than LA in their center. On the other hand, increasing the number of robotic procedures performed per year is an effective depreciation modality of robotic systems and consequently may allow a cost decrease [60]. Indeed, Winter et al. calculated that if a center performs over 500 robotic procedures per year, then capital and maintenance costs for the robotic system would be $380 per procedure [32]. Our analysis is in accordance with these data and further suggests that RAA could become more sustainable in high-volume robotic-surgery centers [26]. Feng et al. [33] recently reported their strategies in order to reduce the cost of RAA, by limiting the number of robotic instruments and energy devices and utilizing an experienced surgical team.The financial model of reimbursement has also an important impact on the choice and feasibility of different techniques and potentially explain the inhomogeneity of literature on the subject of cost of robotic-assisted adrenalectomy.

The present study has the merit of being a case–control, comparative study for robot-assisted and laparoscopic adrenalectomy in a high-volume endocrine referral center, including a large collection of clinical data on minimally invasive LTA. Indeed, inhomogeneity of selection criteria, clinical management, and expertise could represent the main bias source of multicenter studies. The homogeneity of reported data represents one of the strengths of our analysis.

However, this study has several limitations that should be underlined. First, the present series is a retrospective, non-randomized study including patients operated on over a long period. To address the randomization issue, we performed a propensity score to match cases appropriately.

Secondly, the definition of the correct sample size is critical. Indeed, it has been reported that by performing a power analysis, 15,756 patients would be required, in order to achieve a significant difference in terms of operative complications of the two approaches, given the average range of reported complications rate [49]. Lastly, a more detailed subgroup analysis could not be carried out with our current sample size.

In conclusion, outcomes of R-LTA and L-TLA were similar in a selected cohort of patients with adrenal tumors. Moreover, our results underline the potential advantages of the application of the robotic technology to more complex cases (obese patients, hypercortisolism, catecholamines hypersecretion, and large tumor size) in terms of operative time and postoperative complications.

RAA appeared to be cost effective and economically sustainable in a high-volume center (60 adrenalectomies/year), especially if performed in challenging cases, including patients with BMI ≥ 30 kg/m^2^, large (> 6 cm) and/or functioning tumors. However, randomized controlled trials with larger sample sizes are necessary to draw definitive conclusions.

## Supplementary Information

Below is the link to the electronic supplementary material.

Supplementary file1 (DOCX 22 kb)

## Declarations

### Research involving human and/or animals participants

This article does not contain any studies with animals performed by any of the authors.
