# Supplementary material for: Robot-assisted vs laparoscopic lateral transabdominal adrenalectomy: a propensity score matching analysis
Source: Surg Endosc. 2022 Oct 3;36(11):8619–29. doi: 10.1007/s00464-022-09663-3 (PMC9613740; doi:10.1007/s00464-022-09663-3)
Supplement: Supplementary file 1 — Supplementary file1 (DOCX 22 kb) [file 464_2022_9663_MOESM1_ESM.docx]

**Preoperative protocol for patients with catecholamines hypersecretion**. Pharmacologic preparation was performed in all patients with catecholamine-secreting neoplasms at least 7–10 days preoperatively, using alpha-adrenergic or combined alpha- and beta-adrenergic blockade. We also performed a relative volume expansion with approximately 1 L of intravenous ringer lactate at 1 ml/kg/h during the 24 hours prior to surgery.

**Post-operative protocol.** The post-operative patients protocol is described in the supplementary materials. A standard postoperative protocol, optimized for adrenalectomy was used. All patients remained nil per os until postoperative day (POD) 1. Mobilization was initiated the day of the surgery. Postoperative fluid therapy was accomplished with a volume of 1.5 ml/kg/h. One hundred mg intravenous hydrocortisone was administered in case of suspicious acute adrenal insufficiency. Postoperative protocol for corticosteroid therapy in patients with preoperative hypercortisolism is reported in the table.

Routine complete blood examination and blood count were obtained on POD1 in all patients.

The discharge was scheduled at least 24 hours after the surgical procedure when the following conditions were met: no clinical complications or postoperative biochemical and imaging alterations occurred; oral alimentation was tolerated; autonomy in life activities was achieved; the discharge was accepted by the patient; corticosteroid therapy was taken orally.

Routine follow-up with blood test analysis and physical examination was performed one week after hospital discharge. All patients received enoxaparin (4000 UI/0.4 ml) for 10 days, except in cases of hypercortisolism and suspicion for malignancy, where the period was extended to 4 weeks[1].

***Postoperative corticosteroid therapy protocol in patients with hypercortisolism***

| **Post-operative day** | **Corticosteroid therapy** |
| --- | --- |
| **Day 0** | 100 mg IV hydrocortisone every 8 hours after the end of the surgical procedure |
| **Day 1** | 100 mg IV hydrocortisone h 8 + 50 mg IV hydrocortisone h 14 |
| **Day 2** | 75 mg IV hydrocortisone h 8 + 25 mg IV hydrocortisone h 14 |
| **Day 3** | 50 mg IV hydrocortisone h 8 + 20 mg per os hydrocortisone h 14 |
| **Day 4** | 30 mg per os hydrocortisone h 8 + 10 mg per os hydrocortisone h 14 + 10 mg per os hydrocortisone h 18 |
| **Day 5** | 20 mg per os hydrocortisone h 8 + 10 mg per os hydrocortisone h 14 |

IV: Intravenous

***Risk factors for increased operative time in L-TLA (multivariable backward stepwise logistic regression analysis)***

| **Risk factor** | **OR** | **95% CI** | **p-value** |
| --- | --- | --- | --- |
| **Hypercortisolism**  **Lesion Size > 6 cm** | 3.871  4.516 | 0.966 – 15.544  0.876 – 23.280 | **0.041**  **0.048** |

**Supplementary materials references**

1. Babic B, De Roulet A, Volpe A, Nilubol N (2018) Is VTE Prophylaxis Necessary on Discharge for Patients Undergoing Adrenalectomy for Cushing Syndrome? J Endocr Soc 3:304–313. <https://doi.org/10.1210/JS.2018-00278>
